# The genomic bases of atrial fibrillation in an Ecuadorian patient: a case report

**DOI:** 10.3389/fcvm.2025.1552417

**Published:** 2025-06-23

**Authors:** Rafael Tamayo-Trujillo, Patricia Guevara-Ramirez, Leonel Meza-Chico, Santiago Cadena-Ullauri, Viviana A. Ruiz-Pozo, Elius Paz-Cruz, José Luis Laso-Bayas, Rita Ibarra-Castillo, Ana Karina Zambrano

**Affiliations:** ^1^ Universidad UTE, Facultad de Ciencias de la Salud Eugenio Espejo, Centro de Investigación Genética y Genómica, Quito, Ecuador; ^2^ Clinical Cardiac Electrophysiologist, Quito, Ecuador

**Keywords:** case report, healthcare, cardiovascular diseases, genomics, Ecuadorian

## Abstract

Atrial fibrillation (AF) is one of the most globally prevalent arrhythmias with multifactorial factors, including environmental and genetic predisposition influences. The present case report describes a 30-year-old Ecuadorian mestizo male diagnosed with persistent AF with an history of hyperthyroidism, later progressing to hypothyroidism post-radioactive iodine therapy. Genomic test identified variants of uncertain significance in the *TTN*, *MYH11*, and *RAF1* genes, which are associated with cardiovascular diseases but not directly linked to AF. The interplay between thyrotoxicosis and genetic predispositions is discussed as a potential mechanism underlying AF development. This report emphasizes the need for genomic screening and personalized strategies in populations like Ecuador with complex genetic and environmental backgrounds.

## Introduction

Cardiovascular diseases (CVDs) remain the leading cause of mortality worldwide across different ethnic groups (1). In 2019, approximately 17.9 million of deaths were attributed to CVDs, with ischemic heart diseases, and arrhythmias as contributors (2). In Ecuador, CVDs are one of the primary causes of death (3). Atrial fibrillation (AF) is the most common arrhythmia globally, affecting approximately 50 million people as of 2020, with its prevalence increasing due to aging and associated comorbidities (4–6). AF is characterized by uncoordinated atrial electrical activity, resulting to ineffective atrial contractions and hemodynamic compromise (7, 8). It is associated with increased cardiovascular morbidity and mortality, with a 1.5 to 2- fold higher risk of death, and evidence suggests that this risk is grater in women than men (6, 9).

AF is traditionally linked to multifactorial conditions influenced by environmental and structural factors, like cardiac structural diseases, heart valve disease, hypertension, endocrinology diseases as hyperthyroidism and genetic predisposition (10). Heritability studies estimate that up to 30% of AF cases are associated with genetic factors (11). Genome-wide association studies have documented more than 100 loci for AF, some of them consistent in different ethnic groups (11, 12). Genes such as *TTN*, *MYH7*, *MYH6*, *LMNA* and *KCNQ1* playing roles in cardiac structure, ion channel function and cellular signaling pathways (7). Other genes, such as *MYH11*, and *RAF*, may also contribute to arrhythmogenesis through disruption in mechanical and electrical stability.

The Myosin Heavy Chain 11 (*MYH11*) gene encodes a member of the smooth muscle myosin heavy chain family. It functions by converting chemical energy into mechanical energy through the ATP hydrolysis, which is important for cardiac contractility and cytoskeletal integrity. Variants in *MYH11* are inked to structural and arrhythmogenic abnormalities, for its potential disruption in the mechanical and electrical stability (13, 14).

Similarly, Titin (*TTN*) gene encodes a large protein found in striated muscle, providing structural connections at the level of individual microfilaments and balancing sarcomere. Titin also acts as an adhesive element for the contractile machinery in muscle cells. Variants in TTN are associated with arrhythmias caused by sarcomere disfunction (15–18).

Additionally, Raf-1 Proto-Oncogene (*RAF1*) gene encodes MAP 3-kinase, which functions downstream of the Ras family of membrane GTPases to which it binds, regulating the RAS/MAPK pathways. Variants in the gene are implicated in Noonan syndrome, LEOPARD syndrome, hypertrophic cardiomyopathy and electrical instability (19–21).

This report presents the case of an Ecuadorian patient diagnosed with atrial fibrillation. By examining the genetic contribution to AF in this case, the article aims to underscore the importance of genomic screening in understanding the molecular mechanisms of AF, thereby improving diagnosis and management. Such advances in cardiovascular care are critical in Ecuador, where strategies are needed to bridge diagnostic gaps, enhance prevention efforts, and tailor treatments to the unique characteristics of Ecuadorian population. This report adds to the scientific literature by highlighting the importance of genomic and environmental interactions in AF pathogenesis by exploring a unique case from Ecuador, where routine genomic testing is uncommon.

## Case presentation

**Figure 1:**
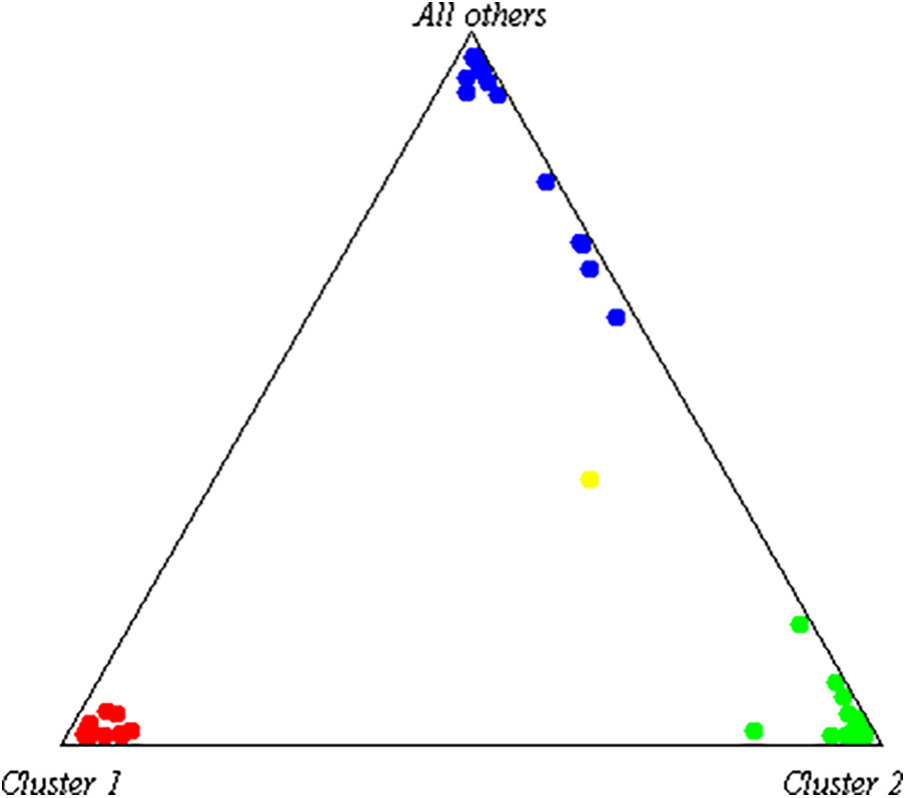
Ancestry composition of the subject. The subject's ancestry is represented in yellow, with African ancestry depicted in red, Native American ancestry in blue, and European ancestry in light green.

**Figure 2:**
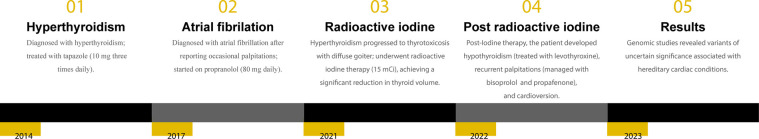
Timeline of clinical events. The figure illustrates the chronological progression of the patient's clinical history, including the diagnosis of hyperthyroidism in 2014, the onset of atrial fibrillation in 2017, radioiodine treatment in 2021, subsequent development of hypothyroidism, genetic testing, and successful electrical cardioversion in 2022 and 2023.

This case study describes a 30-year-old man, self-identified as mestizo, from El Carmen, Manabí, Ecuador. Ancestral component analysis was conducted using 46 Ancestry-Informative Markers (AIMs)-Indels, as described by Zambrano et al. (22), revealing proportions of 34.5% Native American, 47.6% European, and 17.9% African (Figure 1). The study was approved by the UTE University Ethics Committee (CEISH-2021-016), and written informed consent was obtained before performing Next-Generation Sequencing (NGS). The patient also consented to including his clinical and imaging data in scientific publications (Figure 2).

In 2017, the subject was diagnosed with atrial fibrillation (AF) after reporting occasional isolated palpitations. Initial management included propranolol at 80 mg daily. Previously, in 2014, he had been diagnosed with hyperthyroidism and was prescribed tapazole at a dose of 10 mg three times daily. In 2021, his clinical presentation of hyperthyroidism progressed to thyrotoxicosis with diffuse goiter, which showed an enlarged thyroid gland with a thyroid volume of 14.2 ml (male normal value: 4.8–12 ml), Free thyroxine (FT4) of 1.14 ng/dl [Normal value (NV): 0.89–1.76 ng/dl], thyroid-stimulating hormone (TSH) of 2.94 uUI/ml (NV: 0.55–4.76 uUI/ml), and antimicrosomal antibodies (TPO) of 1249.50 UI/ml (NV: <60 UI/ml). Since he did not respond to antithyroid treatment, definitive therapy with radioactive iodine (I-131) was administered at a dose of 15 millicuries (mCi). The procedure was performed without complications and resulted in a significant reduction in thyroid volume. However, subsequent blood tests revealed significantly elevated thyroid autoantibodies, including anti-thyroglobulin (201 UI/ml; Normal Value <60 UI/ml), anti-thyroperoxidase (>6,500 UI/ml; Normal Value <60 UI 7 ml), and abnormal levels of thyroid hormones: FT4 of 0.56 ng/dl, and TSH of 12.65 uUI/ml, consistent with an autoimmune etiology and hypothyroidism following treatment with I-131. The subject developed symptoms of fatigue, lethargy, and constipation, leading to the discontinuation of tapazole, which led to implementing a treatment with oral levothyroxine (LT4) at 75 μg daily, along with recommendations for regular exercise. In four months, he achieved a weight reduction of 5 kg.

However, the proband continued to experience cardiac symptoms, including recurrent episodes of palpitations. As a result, bisoprolol 5 mg daily and propafenone 225 mg three times daily were added to his treatment. His family history revealed no known cardiac diseases; however, his mother had died of pulmonary metastasis, and his maternal and paternal uncles had a history of type 2 diabetes mellitus (Supplementary Figure S1). Moreover, environmental risk factors included exposure to ultraviolet radiation daily, and pesticides twice a week during his eight years of work in agricultural fields.

In 2022, one year after iodine therapy, the subject presented with chest tightness, which culminated in syncope accompanied by sphincter relaxation. Given this condition, an electrocardiogram (EKG) was performed, and no dynamic alterations of the T wave or ST segment suggestive of myocardial ischemia were detected. However, there was evidence of irregularities in the R-R interval and an absence of P waves confirming atrial fibrillation rhythm.

The patient was admitted to the cardiology department, where Holter monitoring confirmed persistent atrial fibrillation. During hospitalization, a presumptive diagnosis of systemic connective tissue involvement was considered, with suspicion of an autoimmune condition potentially underlying the arrhythmia. A rheumatology consultation was requested, but tests for antinuclear antibodies (ANA) and anti-DNA antibodies were negative.

One month after admission, an echocardiogram was performed to evaluate his left atrium and rule out thrombi, facilitating the safe execution of electrical cardioversion. The procedure was successfully conducted using a synchronized 200-joule shock, without complications.

## Follow-up and outcomes

In 2023, it was suggested to investigate the presence of genomic variants associated with hereditary cardiac conditions. In this context, genomic studies were performed using next-generation sequencing. The patient's genomic DNA was analyzed with the TruSight Cardio (TSC) panel (Illumina, Inc. San Diego, CA, USA), which includes 174 genes associated with 17 cardiac conditions. The sequencing was conducted on the Illumina MiSeq platform, achieving a coverage of ≥20× in 97.91% of the targeted regions. The results revealed that the proband is heterozygous for a variant of uncertain significance (classified according to ACMG guidelines (23), NM 001267550.2:c.97442G>A:p.(Gly 32481Glu), in the *TTN* gene. Additionally, two other variants of uncertain significance (classified according to ACMG guidelines (23) were identified: *MYH11* gene variant NM_002474.2:c.2288T>C: p.(Ile763Thr) and *RAF1* gene variant NM_002880.3:c.37A>G:p. (Asn13Asp). These variants are associated with cardiovascular diseases and could potentially explain the patient's persistent atrial fibrillation (Table 1).

**Table 1:** Genetic variant detected in the patient.

Gene	Variant type	NCBI references sequence: nucleotide/protein change	dbSNP	Suggested classification (ACMG)	ACMG classification	Frequency
*TTN*	Missense variant	NM 001267550.2:c.97442G > A:p.(Gly 32481Glu)	rs201364164	Uncertain significance	PM2, BP6^a^	ALFA: 0.000^b^
*MYH11*	Missense variant	NM_002474.2:c.2288T > C: p.(Ile763Thr)	rs944017240	Uncertain significance	PM2, PP3^a^	ALFA: 0.000^b^
*RAF1*	Missense variant	NM_002880.3:c.37A > G:p. (Asn13Asp)	rs1382771046	Uncertain significance	PM2^a^	ALFA: 0.000^b^

^a^ PM2: Absent from controls (or at extremely low frequency if recessive) in Exome Sequencing Project, 1,000 Genomes Project, or Exome Aggregation Consortium; BP6: Reputable source recently reports variant as benign, but the evidence is not available to the laboratory to perform an independent evaluation; PP3: Multiple lines of computational evidence support a deleterious effect on the gene or gene product.

^b^ Frequency for the Latin American population from the ALFA project.

At the patient's most recent follow-up, a sinus rhythm was observed on the ECG, with no irregularities in the R-R interval characteristic of atrial fibrillation. Dynamic alterations in the T wave suggestive of ischemia or prolongation of the PR interval (0.20 s) were also absent, indicating a favorable response to the treatment administered. However, mild ST-segment elevations in lead D2 were observed, likely due to the pharmacological effects of propafenone.

**Figure 3:**
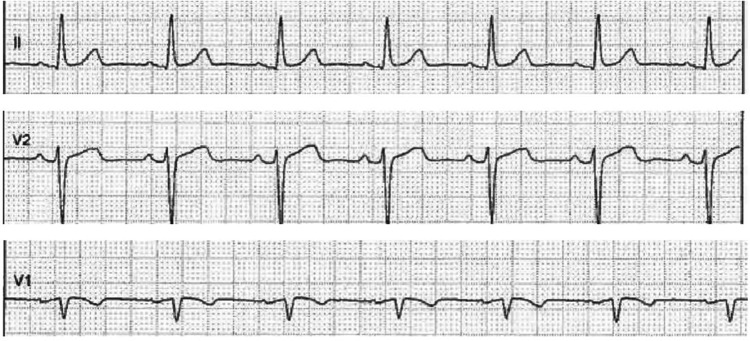
Patient's electrocardiogram. Left atrial enlargement, likely secondary to atrial fibrillation, is evidenced by a broad terminal *P* wave (>0.12 s) in leads V1 and V2 and a notched P wave in lead D2, indicating left atrial hypertrophy.

Other findings in the ECG included a negative T wave in lead V1, which may be normal in certain young patients, however, due to the proband's arrhythmia we can suspect an abnormal ventricular repolarization. Additionally, permanent morphological changes consistent with left atrial enlargement were noted. These changes were likely attributable to the atrial fibrillation history and were evident in leads V1 and V2, where a P wave with a terminal component exceeding 0.12 s was observed. Moreover, a notched P wave in lead D2 further supported the diagnosis of left atrial hypertrophy due to chronic atrial fibrillation (Figure 3).

## Discussion

Atrial fibrillation (AF) has been associated with thyroid dysfunction, which leads to an increased risk of morbidity and mortality (24, 25). Thyroid hormones regulate the expression of several genes involved in the generation and conduction of ionic currents in the heart (25). Moreover, it has been described that hyperthyroidism or hypothyroidism was related to atrial arrhythmia and ventricular arrhythmia, respectively (26). Furthermore, an altered thyroid profile could promote a cardiac evaluation in individuals without genetic or congenital predisposition to cardiac arrhythmias. Therefore, early pharmacological treatment of hypo/hyperthyroidism symptomatology could improve or prevent future cardiac events (26).

In this case, a young individual without genomic variants strongly related to AF and a clear hyperthyroidism profile showed a persistent AF phenotype under beta-blocker, antiarrhythmic, and antithyroid pharmacological treatments. It has been described that hyperthyroidism is associated with a shortened action potential duration, which triggers the development of AF (27). Moreover, the pharmacological treatment of hyperthyroidism was related to an improved heartbeat; however, a persistent AF under radioactive iodine treatment has also been observed, which was related to thyrotoxicosis (high triiodothyronine levels in blood), history of smoking, and older age (28, 29). There was no history of smoking in this patient, however, a large period of exposure to pesticides (8 years) was reported in his clinal record. This exposure could trigger the thyrotoxicosis and the subsequent AF. Moreover, high blood levels of antithyroglobulin and antimicrosomal antibodies suggest an autoimmune thyroid disease as the trigger for thyrotoxicosis. These approaches could be evaluated with a specific genomic panel to autoimmune diseases to discard Grave's Disease.

In addition to thyrotoxicosis, several genetic variants related to cardiac alterations were detected. First, a missense variant p.(Ile763Thr) in the *MYH11* gene was detected. This mutation has not been previously related to AF; however, it has been related to familial thoracic aortic aneurysm (30). For instance, Pucci et al., identified MYH11 variants in a family with Thoracic aortic aneurysms and syncope episodes (31). Similarly, Petrea et al., described a MYH11 genetic variant in a patient with intracranial aneurism, aortic abnormalities, and family history of stroke (32). Even though, this patient did not present aneurysms, this genetic variant must be considered to promote surveillance for the potential emergence of aneurysms or syncope, as previously reported in the proband. This surveillance must be permanent due to the autosomal dominant inheritance of this disease, and the reports of aortic disease (33, 34) and intracranial vessel stenosis (35), related to mutations in this gene. Like *MYH11*, *MYH6* and *MYH7* also encode for the molecular motor's myosin heavy chain alpha and beta, respectively (36), and variations in these genes have been associated with reduced cardiac performance and higher occurrence of AF (37). Therefore, the *MYH11* variant detected in this proband could also contribute to less cardiac structural integrity, which could lead to the early onset of AF as described in this case. However, the underlying molecular mechanism remains unclear and could be further evaluated to describe its role in AF pathophysiology.

Another missense variant p.(Asn13Asp) in the *RAF1* gene was also identified. This variant has not been previously correlated with AF. However, a variant in this gene (p.Ser257 Leu) was related to various types of arrhythmias including AF, in a newborn male with Noonan syndrome. In this case, the underlying mechanism of AF was associated with an impaired calcium signaling caused by the activation of the calcineurin/nuclear factor of activated T cells and downregulation of sarco/endoplasmic reticulum Ca2^+^-ATPase 2a (38). Moreover, dilated cardiomyopathy (DCM), a disease with an autosomal dominant inheritance pattern, and sudden cardiac death have been associated with mutations in this gene. The echocardiogram results of this patient did not show any structural abnormalities. However, active surveillance must be carried out to prevent cardiac complications in the future (39). Therefore, it is crucial to understand the role of *RAF1* variants in AF pathophysiology through cardiac electrophysiology alterations given the association between *RAF1* variants and ventricular arrhythmias (40).

Lastly, another missense variant p.(Gly32481Glu) in the *TTN* gene was also detected. Notably, variants in this gene were not related to AF; however, *TTN* variants have been previously correlated with cardiac disease. For instance, Paz-Cruz et al., identified p.(Val23881Leu) and p.(Arg3224Gly) variants in a 43-year-old man with an episode of aborted sudden cardiac death and diagnosed with a cardiac arrhythmia (41). Similarly, another *TTN* variant (c.6790+3 A>G) was correlated with exertional dyspnea and DCM in a 32-year-old man (42).

Loss of function variants in this gene have been associated with abnormal sarcomere function, atrial fibrosis, and longer PR interval, which were related to AF pathogenesis in zebra fish models (43). Furthermore, a case-control study showed that impaired sarcomere structure and function were related to *TTN* variants and suggested its role in early-onset AF (44). Mutations in this gene have also been correlated with DCM, which could potentially trigger heart failure, sudden cardiac death, and the need for a heart transplant (45). The inheritance form of DCM is also autosomal dominant, although not all variants are disease-causing (46). In this case, a normal dimension of the heart in the patient's clinical record could suggest that this variant does not cause DCM; however, surveillance is advised due to the risk of potentially developing cardiomyopathies. Therefore, the *TTN* variant described in this case could be implicated in the proband's early-onset AF. Further research is required to understand the underlying mechanisms of *TTN* and cardiac disease.

Therefore, the identification of novel genetic variants in *MYH11*, *RAF1*, and *TTN* genes in a patient with early-onset AF, with absence of structural cardiac anomalies, highlights the potential role of this variants in the pathogenesis of AF. Taken together, variants in *MYH11*, *RAF1*, and *TTN* genes could be implicated in an altered structural integrity of cardiac muscle, electrophysiological, sarcomere integrity, and atrial fibrosis, which may underlie the early onset of AF, as it has been observed in this proband. These findings underscore the role of genetic screening in individuals with unexplained AF that may reveal genetic risk factors correlated with AF, prognosis, and personalized management strategies.

The ancestral analysis of this patient showed a high European proportion (47.6%) and a low African proportion (17.9%). It has been reported that Black Americans have more risk of developing hyperthyroidism (47). Moreover, Grave's Disease is also more common in black people than white (48, 49). The predisposition to this pathology could be evaluated in mestizo populations like the Ecuadorian due to the high African descendant people present in this country (22). Therefore, the association between ancestry proportion, genomic variants, and clinical phenotype could improve the diagnosis challenges and implement a personalized therapeutic strategy.

The novelty of the present case report lies in the comprehensive approach utilized. This study not only presents a clinical evaluation but also incorporates an environmental history, identifying potential detrimental effects of chronic pesticide exposure that may have contributed to the patient's cardiac pathology. In addition, the report considers pharmacological treatments that may have exacerbated the cardiac condition in the participant.

The study also includes genetic ancestry analysis in an underrepresented population, such as that of Ecuador. Due to the intricate country's history of colonization and admixture processes, the genetic architecture of its population is particularly complex, as seen in the participant. The patient's ancestry shows a predominantly European ancestry background, followed by Native American and African components. These findings highlight the importance of considering genetic ancestry in the clinical interpretation of disease risk and presentation.

Furthermore, genetic analysis revealed variants that could explain the cardiac phenotype of the patient. The possibility of additive effects between these variants could increase their potential pathogenicity. However, further research, such as in-silico and functional studies, are needed to clarify the impact of these variants and how they may be related to the patient's pathology.

The present case report has limitations. For instance, there is a lack of similar case reports studies that correlates the role of *MYH11*, *TTN* or *RAF1* variants and AF. Moreover, there is a limited number of genomic tests to detect variants associated with autoimmune diseases, which give the clinical phenotype of this patient, may potentially lead to a precise diagnosis and tailored treatment.

Furthermore, there is no genomic data of the proband's family that could help to determine if the detected variants were inherited or not. Although the clinical record showed type 2 Diabetes and cancer in the patient's relatives, no records of autoimmune or cardiomyopathies were present. Due to the patient's environmental exposure to pesticides, it is important to investigate the potential connection between this toxic exposure and the development of thyrotoxicosis in this case. This exploration could help identify genetic variants that make individuals more susceptible to thyrotoxicosis when exposed to environmental stressors like pesticides. Additionally, recent findings have linked genetically influenced behavioral factors, such as smoking initiation, to an increased risk of atrial fibrillation (50). Although the patient has no history of smoking, these findings emphasize the need to examine gene-environment interactions in complex diseases like atrial fibrillation and thyrotoxicosis.

A significant limitation of this report is the lack of regional genomic data, which constrains the interpretation of the identified variant of VUS. Although the variants exhibit an allele frequency of zero in Latino populations according to ALFA, the absence of population-specific reference datasets from Ecuador or Latin America limits the ability to contextualize their clinical relevance. This highlights the urgent need to expand genetic databases in underrepresented populations to enhance the accuracy of variant classification.

Finally, another important limitation of this case is the lack of long-term follow-up data. Clinical outcomes, including the recurrence of atrial fibrillation or the progression of structural heart disease, could not be evaluated. Although limitations in healthcare access hindered long-term follow-up, such monitoring is crucial for better understanding the prognostic impact of the identified genetic variants and environmental exposures. Future studies should include long-term clinical follow-up to validate the pathogenic relevance and clinical significance of these findings.

## Conclusion

This case highlights the multifactorial risk factors of AF in a young individual with hyperthyroidism and no genomic variants related to AF. Persistent AF under pharmacological and radioactive iodine treatments, stand out the complex interplay of thyrotoxicosis, pesticide exposure, and AF development. This interplay could promote future research that explores the role of pesticide exposure in the development of thyrotoxicosis and cardiac pathologies.

## Data Availability

The datasets generated and/or analyzed during the current study are available in the Sequence Read Archive (SRA) repository, BioProject number PRJNA1276548. For more information, please contact the corresponding author AZ (anazambrano17@hotmail.com).

## Supplementary material

Supplementary Figure 1 **Pedigree of the Family.** The pedigree outlines the family history across three generations (I, II, III). While the proband presents with arrhythmia, no similar conditions are observed in other family members. Notable familial history includes diabetes mellitus in maternal and paternal uncles and pulmonary metastasis in the proband's mother.

## References

[1] Zambrano AK Cadena-Ullauri S Guevara-Ramírez P Paz-Cruz E Tamayo-Trujillo R Ruiz-Pozo VA The autosomal short tandem repeat polymorphisms are potentially associated with cardiovascular disease predisposition in the Latin American population: a Mini review. Biomed Res Int. (2023) 2023:6152905. 10.1155/2023/6152905 38027043 PMC10651335

[2] World Health Organization Cardiovascular Diseases (CVDs). Available at: https://www.who.int/news-room/fact-sheets/detail/cardiovascular-diseases-(cvds)?gad_source=1&gclid=Cj0KCQiAsaS7BhDPARIsAAX5cSAy4k3zz3mzsPoBg2564Ywt6X9GWvzb8qnyrH0UgvDS3o16ZLdqS34aAlBmEALw_wcB (Accessed December 22, 2024).

[3] INEC Estadísticas Vitales. Available at: https://www.ecuadorencifras.gob.ec/documentos/web-inec/Poblacion_y_Demografia/Defunciones_Generales/2023/Principales_resultados_EDG_2023.pdf (Accessed May 25, 2025).

[4] Tsao CW Aday AW Almarzooq ZI Anderson CAM Arora P Avery CL Heart disease and stroke statistics—2023 update: a report from the American Heart Association. Circulation. (2023) 147:E93–E621. 10.1161/CIR.0000000000001123/FORMAT/EPUB 36695182 PMC12135016

[5] Schnabel RB Yin X Gona P Larson MG Beiser AS McManus DD Fifty-year trends in atrial fibrillation prevalence, incidence, risk factors, and mortality in the community. Lancet. (2015) 386:154. 10.1016/S0140-6736(14)61774-8 25960110 PMC4553037

[6] Joglar JA Chung MK Armbruster AL Benjamin EJ Chyou JY Cronin EM 2023 ACC/AHA/ACCP/HRS guideline for the diagnosis and management of atrial fibrillation: a report of the American College of Cardiology/American Heart Association joint committee on clinical practice guidelines. Circulation. (2024) 149:E1–E156. 10.1016/j.jacc.2023.08.017 38033089 PMC11095842

[7] Hindricks G Potpara T Kirchhof P Kühne M Ahlsson A Balsam P 2020 ESC guidelines for the diagnosis and management of atrial fibrillation developed in collaboration with the European Association for Cardio-Thoracic Surgery (EACTS): the Task Force for the diagnosis and management of atrial fibrillation of the European Society of Cardiology (ESC) developed with the special contribution of the European Heart Rhythm Association (EHRA) of the ESC. Eur Heart J. (2021) 42(5):373–498. 10.1093/eurheartj/ehaa612 32860505

[8] Tamayo-Trujillo R Paz-Cruz E Cadena-Ullauri S Guevara-Ramirez P Ruiz-Pozo VA Ibarra-Castillo R Exploring atrial fibrillation: understanding the Complex relation between lifestyle and genetic factors. J Med Cases. (2024) 15:186–94. 10.14740/JMC4250 39091575 PMC11287905

[9] Emdin CA Wong CX Hsiao AJ Altman DG Peters SAE Woodward M Atrial fibrillation as risk factor for cardiovascular disease and death in women compared with men: systematic review and meta-analysis of cohort studies. Br Med J. (2016) 532:1–10. 10.1136/BMJ.H7013 PMC5482349 26786546

[10] Frost L Vestergaard P Mosekilde L. Hyperthyroidism and risk of atrial fibrillation or flutter: a population-based study. Arch Intern Med. (2004) 164:1675–8. 10.1001/ARCHINTE.164.15.1675 15302638

[11] Roselli C Chaffin MD Weng LC Aeschbacher S Ahlberg G Albert CM Multi-Ethnic genome-wide association study for atrial fibrillation. Nat Genet. (2018) 50:1225–33. 10.1038/s41588-018-0133-9 29892015 PMC6136836

[12] Weng LC Choi SH Klarin D Smith JG Loh PR Chaffin M Heritability of atrial fibrillation. Circ Cardiovasc Genet. (2017) 10:1–7. 10.1161/CIRCGENETICS.117.001838 PMC5966046 29237688

[13] Zhu L Vranckx R Van Kien PK Lalande A Boisset N Mathieu F Mutations in myosin heavy chain 11 cause a syndrome associating thoracic aortic aneurysm/aortic dissection and patent ductus arteriosus. Nat Genet. (2006) 38:343–9. 10.1038/ng1721 16444274

[14] GeneCards MYH11 Gene. Available at: https://www.genecards.org/cgi-bin/carddisp.pl?gene=MYH11 (Accessed December 22, 2024).

[15] Lange S Xiang F Yakovenko A Vihola A Hackman P Rostkova E Cell biology: the kinase domain of titin controls muscle gene expression and protein turnover. Science. (2005) 308:1599–603. 10.1126/science.1110463 15802564

[16] Itoh-Satoh M Hayashi T Nishi H Koga Y Arimura T Koyanagi T Titin mutations as the molecular basis for dilated cardiomyopathy. Biochem Biophys Res Commun. (2002) 291:385–93. 10.1006/bbrc.2002.6448 11846417

[17] GeneCards TTN Gene. Available at: https://www.genecards.org/cgi-bin/carddisp.pl?gene=TTN&keywords=ttn (Accessed December 22, 2024).

[18] Gerull B Gramlich M Atherton J McNabb M Trombitás K Sasse-Klaassen S Mutations of TTN, encoding the giant muscle filament titin, cause familial dilated cardiomyopathy. Nat Genet. (2002) 30:201–4. 10.1038/ng815 11788824

[19] Pandit B Sarkozy A Pennacchio LA Carta C Oishi K Martinelli S Gain-of-function RAF1 mutations cause noonan and LEOPARD syndromes with hypertrophic cardiomyopathy. Nat Genet. (2007) 39:1007–12. 10.1038/ng2073 17603483

[20] Kobayashi T Aoki Y Niihori T Cavé H Verloes A Okamoto N Molecular and clinical analysis of RAF1 in noonan syndrome and related disorders: dephosphorylation of serine 259 as the essential mechanism for mutant activation. Hum Mutat. (2010) 31:284–94. 10.1002/humu.21187 20052757

[21] GeneCards RAF1 Gene. Available at: https://www.genecards.org/cgi-bin/carddisp.pl?gene=RAF1&keywords=raf1 (Accessed December 22, 2024).

[22] Zambrano AK Gaviria A Cobos-Navarrete S Gruezo C Rodríguez-Pollit C Armendáriz-Castillo I The three-hybrid genetic composition of an Ecuadorian population using AIMs-InDels compared with autosomes, mitochondrial DNA and Y chromosome data. Sci Rep. (2019) 9:1–8. 10.1038/S41598-019-45723-W 31239502 PMC6592923

[23] Richards S Aziz N Bale S Bick D Das S Gastier-Foster J Standards and guidelines for the interpretation of sequence variants: a joint consensus recommendation of the American college of medical genetics and genomics and the association for molecular pathology. Genet Med. (2015) 17:405–24. 10.1038/GIM.2015.30 25741868 PMC4544753

[24] Li R Bin; Yang XH Zhang JD Wang D Cui XR Bai L The association between subclinical thyroid dysfunction and recurrence of atrial fibrillation after catheter ablation. Front Cardiovasc Med. (2022) 9:902411. 10.3389/FCVM.2022.902411/BIBTEX 35722102 PMC9203885

[25] Müller P Leow MKS Dietrich JW. Minor perturbations of thyroid homeostasis and Major cardiovascular endpoints—physiological mechanisms and clinical evidence. Front Cardiovasc Med. (2022) 9:942971. 10.3389/FCVM.2022.942971/BIBTEX 36046184 PMC9420854

[26] Marrakchi S Kanoun F Idriss S Kammoun I Kachboura S. Arrhythmia and thyroid dysfunction. Herz. (2015) 40(Suppl 2):101–9. 10.1007/S00059-014-4123-0 24990773

[27] Bielecka-Dabrowa A Mikhailidis DP Rysz J Banach M. The mechanisms of atrial fibrillation in hyperthyroidism. Thyroid Res. (2009) 2:4. 10.1186/1756-6614-2-4 19341475 PMC2680813

[28] Zhou ZH Ma LL Wang LX. Risk factors for persistent atrial fibrillation following successful hyperthyroidism treatment with radioiodine therapy. Intern Med. (2011) 50:2947–51. 10.2169/INTERNALMEDICINE.50.6135 22185984

[29] Wong CL Tam HKV Fok CKV Lam PKE Fung LM. Thyrotoxic atrial fibrillation: factors associated with persistence and risk of ischemic stroke. J Thyroid Res. (2017) 2017:4259183. 10.1155/2017/4259183 29379659 PMC5742874

[30] National Center for Biotechnology Information ClinVar. [VCV000922759.20] Available at: https://www.ncbi.nlm.nih.gov/clinvar/variation/922759/?oq=((913457[AlleleID]))&m=NM_002474.3(MYH11):c.2288T%3EC%20(p.Ile763Thr) (Accessed December 22, 2024).

[31] Pucci L Pointet A Good JM Davoine E Cina V Zanchi F A new variant in the MYH11 gene in a familial case of thoracic aortic aneurysm. Ann Thorac Surg. (2020) 109:e279–81. 10.1016/J.ATHORACSUR.2019.07.030 31473177

[32] Petrea RE Frank NY Olifir O Siegel CD Huggins H Babikian VL. A rare MYH11 gene missense variant in a patient with intracerebral vascular and ascending aortic disease and clinical strokes (P3-14.004). Neurology. (2025) 104:3–14. 10.1212/WNL.0000000000212275

[33] Kwartler C Kuang S-Q Prakash S Byanova K Pham J Huang J Abstract 67: a rare variant in Myh11, R247C, affects smooth muscle cell phenotype and acts as a modifier for aortic disease. In: Freedman JE, editor. Proceedings of the Circulation Research. (vol. 11). United states: Lippincott Williams & Wilkins (2012). p. A67.

[34] Kuang SQ Kwartler CS Byanova KL Pham J Gong L Prakash SK Rare, nonsynonymous variant in the smooth muscle-specific isoform of myosin heavy chain, MYH11, R247C, alters force generation in the aorta and phenotype of smooth muscle cells. Circ Res. (2012) 110:1411–22. 10.1161/CIRCRESAHA.111.261743/-/DC1 22511748 PMC3917690

[35] Larson A Rinaldo L Brinjikji W Klaas J Lanzino G. Intracranial vessel stenosis in a young patient with an MYH11 mutation: a case report and review of 2 prior cases. World Neurosurg. (2020) 137:243–6. 10.1016/J.WNEU.2020.02.054 32081817

[36] Nielsen JB Thorolfsdottir RB Fritsche LG Zhou W Skov MW Graham SE Biobank-driven genomic discovery yields new insight into atrial fibrillation biology. Nat Genet. (2018) 50:1234. 10.1038/S41588-018-0171-3 30061737 PMC6530775

[37] Andersen JH Andreasen L Olesen MS. Atrial fibrillation—a complex polygenetic disease. Eur J Hum Genet. (2021) 29:1051–60. 10.1038/s41431-020-00784-8 33279945 PMC8298566

[38] Hagino M Ota C Onoki T Iwasawa S. Male infant with noonan syndrome with RAF-1 gene mutation who survived hypertrophic cardiomyopathy-induced fatal heart failure and uncontrollable arrhythmias. BMJ Case Reports CP. (2022) 15:e250342. 10.1136/BCR-2022-250342 PMC9528629 36171012

[39] Zheng J Peng L Cheng R Li Z Xie J Huang E RAF1 mutation leading to hypertrophic cardiomyopathy in a Chinese family with a history of sudden cardiac death: a diagnostic insight into noonan syndrome. Mol Genet Genomic Med. (2023) 12:e2290. 10.1002/MGG3.2290 37787490 PMC10767430

[40] Fagarsan A Al Hussein H Ghiragosian Rusu SE. RAF-1 mutation associated with a risk for ventricular arrhythmias in a child with noonan syndrome and cardiovascular pathology. J Crit Care Med. (2022) 8:126. 10.2478/JCCM-2022-0007 PMC9097645 35950157

[41] Paz-Cruz E Ruiz-Pozo VA Cadena-Ullauri S Guevara-Ramirez P Tamayo-Trujillo R Ibarra-Castillo R Associations of MYPN, TTN, SCN5A, MYO6 and ELN mutations with arrhythmias and subsequent sudden cardiac death: a case report of an Ecuadorian individual. Cardiol Res. (2023) 14:409. 10.14740/CR1552 37936622 PMC10627373

[42] Han S Zhang YY Geng J. Case report: a novel variant of the TTN gene and two other rare variants in a Chinese patient with dilated cardiomyopathy. Front Cardiovasc Med. (2025) 12:1527544. 10.3389/FCVM.2025.1527544/BIBTEX 40271130 PMC12014737

[43] Ahlberg G Refsgaard L Lundegaard PR Andreasen L Ranthe MF Linscheid N Rare truncating variants in the sarcomeric protein titin associate with familial and early-onset atrial fibrillation. Nat Commun. (2018) 9:1–11. 10.1038/s41467-018-06618-y 30333491 PMC6193003

[44] Choi SH Weng LC Roselli C Lin H Haggerty CM Shoemaker MB Association between titin loss-of-function variants and early-onset atrial fibrillation. JAMA. (2018) 320:2354. 10.1001/JAMA.2018.18179 30535219 PMC6436530

[45] Tharp CA Haywood ME Sbaizero O Taylor MRG Mestroni L. The giant protein titin’s role in cardiomyopathy: genetic, transcriptional, and post-translational modifications of TTN and their contribution to cardiac disease. Front Physiol. (2019) 10:494019. 10.3389/FPHYS.2019.01436/BIBTEX PMC6892752 31849696

[46] Jolfayi AG Kohansal E Ghasemi S Naderi N Hesami M MozafaryBazargany MH Exploring TTN variants as genetic insights into cardiomyopathy pathogenesis and potential emerging clues to molecular mechanisms in cardiomyopathies. Sci Rep. (2024) 14:5313. 10.1038/S41598-024-56154-7 38438525 PMC10912352

[47] Chen J Zhang L Zhang X. Overall, sex-and race/ethnicity-specific prevalence of thyroid dysfunction in US adolescents aged 12–18 years. Front Public Health. (2024) 12:1366485. 10.3389/FPUBH.2024.1366485/BIBTEX 38966695 PMC11222593

[48] Antonelli A Ferrari SM Ragusa F Elia G Paparo SR Ruffilli I Graves’ disease: epidemiology, genetic and environmental risk factors and viruses. Best Pract Res Clin Endocrinol Metab. (2020) 34:101387. 10.1016/J.BEEM.2020.101387 32107168

[49] Grixti L Lane LC Pearce SH. The genetics of Graves’ disease. Rev Endocr Metab Disord. (2024) 25:203–14. 10.1007/S11154-023-09848-8/FIGURES/2 38108994 PMC10808215

[50] Wu Z Tang C Wang D. Bidirectional two-sample Mendelian randomization study of association between smoking initiation and atrial fibrillation. Tob Induc Dis. (2024) 22:10.18332/tid/189380. 10.18332/TID/189380 PMC11186308 38899119

