# Supplementary figures and images for: The genomic bases of atrial fibrillation in an Ecuadorian patient: a case report
Source: Front Cardiovasc Med. 2025 Jun 23;12:1552417. doi: 10.3389/fcvm.2025.1552417 (PMC12229997; doi:10.3389/fcvm.2025.1552417)

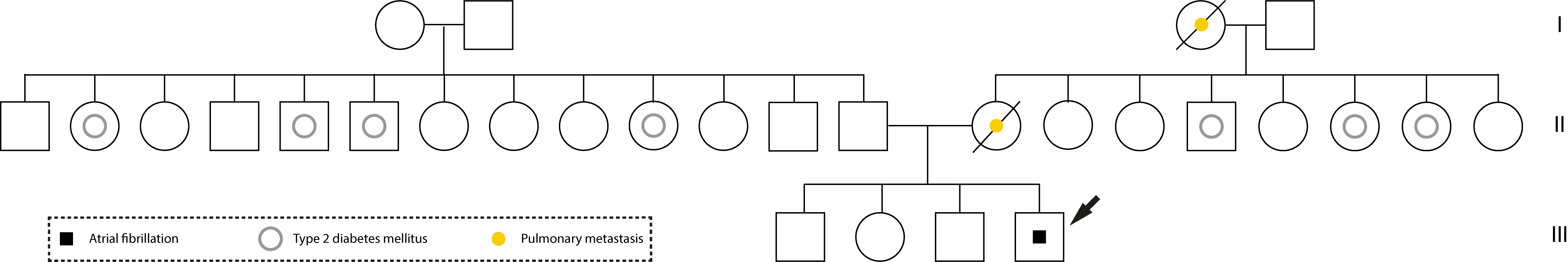

Supplement: Supplementary Figure 1 — Pedigree of the Family. The pedigree outlines the family history across three generations (I, II, III). While the proband presents with arrhythmia, no similar conditions are observed in other family members. Notable familial history includes diabetes mellitus in maternal and paternal uncles and pulmonary metastasis in the proband's mother. [file Image1.tiff]
